# Isolated Traumatic Bitemporal Hemianopia

**DOI:** 10.7759/cureus.17593

**Published:** 2021-08-31

**Authors:** Shazana Ahmad Fauzi, Yusof Nor Sharina, Mohtar Ibrahim

**Affiliations:** 1 Department of Ophthalmology and Visual Science, School of Medical Sciences Universiti Sains Malaysia, Kubang Kerian, MYS; 2 Department of Ophthalmology, Hospital Raja Perempuan Zainab II, Kota Bharu, MYS

**Keywords:** bitemporal hemianopia, optic chiasm, severe head injury, traumatic chiasmal injury, traumatic chiasmal syndrome

## Abstract

Bitemporal hemianopia is a rare presentation following severe head injury, which causes a delay in detection of the visual symptoms. This is a case of traumatic bitemporal hemianopia in a 20-year-old gentleman after an alleged motorbike accident. He was intubated following a severe head injury. The ophthalmologic assessment was performed after he regained consciousness and complained of acute blurring of vision in bilateral eyes. The visual acuity was 2/60 on the right eye and 4/60 on the left eye. Bilateral eyes anterior and posterior segments examination were unremarkable. Confrontation visual field testing discovered bitemporal hemianopia. Plain computed tomography (CT) scan of the brain showed right frontal bone fracture extending to the right orbital roof, superomedial wall of the right orbit, bilateral lamina papyracea, ethmoidal air cells, roof and bilateral walls of sphenoid sinus. He was treated conservatively by the ophthalmology team. Subsequent follow-ups showed improvement of visual acuity which were 6/6 on the right eye and 6/6 on the left eye. Humphrey visual field test confirmed the persistence of bitemporal hemianopia. His good visual acuity does not correlate with the severity of the field defect. Therefore, surveillance for bitemporal scotoma is necessary for all head injuries with severe midline facial bone fractures.

## Introduction

Traumatic chiasmal syndrome is an uncommon phenomenon that presents with bitemporal hemianopia seen after severe head trauma. It was first reported by Nieden in 1883. Such cases are rarely reported since the majority of the patients had prolonged duration of unconsciousness and poor survival as a result of severe head trauma complications [1,2].

Traumatic chiasmal syndrome can occur alone or in conjunction with other neurological symptoms indicating damage to other structures in the anterior cranial fossa or at the base of the skull [2-4]. The visual acuity may remain normal in the presence of significant bitemporal hemianopia after optic chiasm trauma.

The presentation in our case is quite similar to previous reports but infrequently reported in Malaysia. We describe a case of isolated traumatic chiasmal syndrome resulting from significant brain injury without other endocrine or neurological deficits.

## Case presentation

A 20-year-old healthy gentleman was allegedly involved in a motorbike accident and sustained head trauma. He was brought to us semi-conscious with Glasgow Coma Scale E1V2M5. Pupils were equal and reactive. He was intubated for cerebral protection. Upon regaining consciousness, he complained of painless blurred vision in both eyes. His premorbid vision was good without glasses.

Full ophthalmologic examination was performed eight days after trauma revealed the visual acuity of 2/60 on the right eye and 4/60 on the left eye. Pupils were equal in size and reactive without relative afferent pupillary defects. There was a limitation of elevation and depression on the right eye associated with mechanical ptosis due to periorbital hematoma. Bitemporal scotoma was elicited by confrontation. Otherwise, bilateral eyes anterior and posterior segments examination were unremarkable. Systemic examination showed multiple facial abrasion wounds and skin bruises without significant injuries elsewhere. The differential diagnosis was made as bitemporal hemianopia secondary to severe traumatic brain injury.

**Figure 1:**
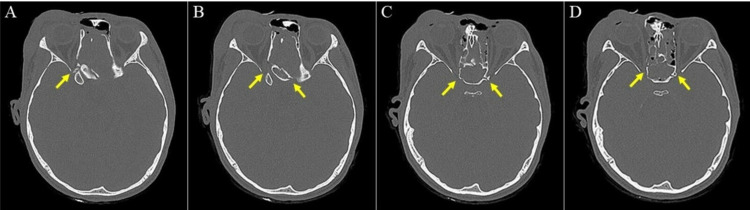
Serial plain CT images of brain and orbit. Arrows showing the oblique fracture line involving the superomedial wall of the right orbit, roof and bilateral walls of sphenoid sinus. CT: computed tomography.

**Figure 2:**
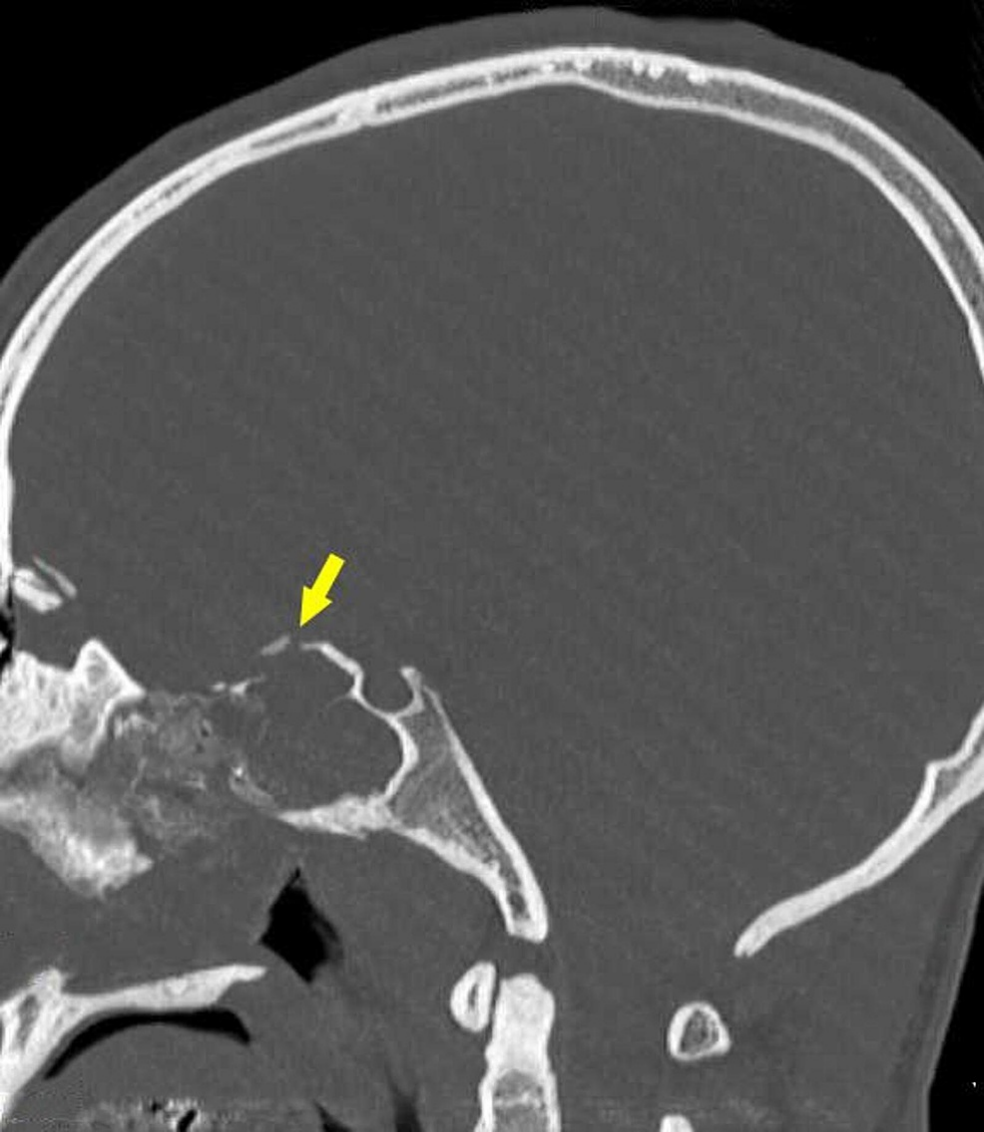
Sagittal CT image showing fracture of the roof of the sphenoid sinus. Arrow shows discontinuity and displacement of bone at the roof of sphenoid sinus indicating fracture of the roof of sphenoid sinus. CT: computed tomography.

Initial plain computed tomography (CT) scan showed right frontal bone fracture with fracture line seen extending posteriorly in an oblique direction across the right orbital roof, superomedial wall of the right orbit, bilateral lamina papyracea, ethmoidal air cells, roof and bilateral walls of sphenoid sinus (Figure 1, 2).

There was also the presence of extradural, subarachnoid and intraparenchymal hemorrhages at the right frontal region causing focal cerebral edema. He was diagnosed with traumatic chiasmal syndrome and severe traumatic brain injury.

**Figure 3:**
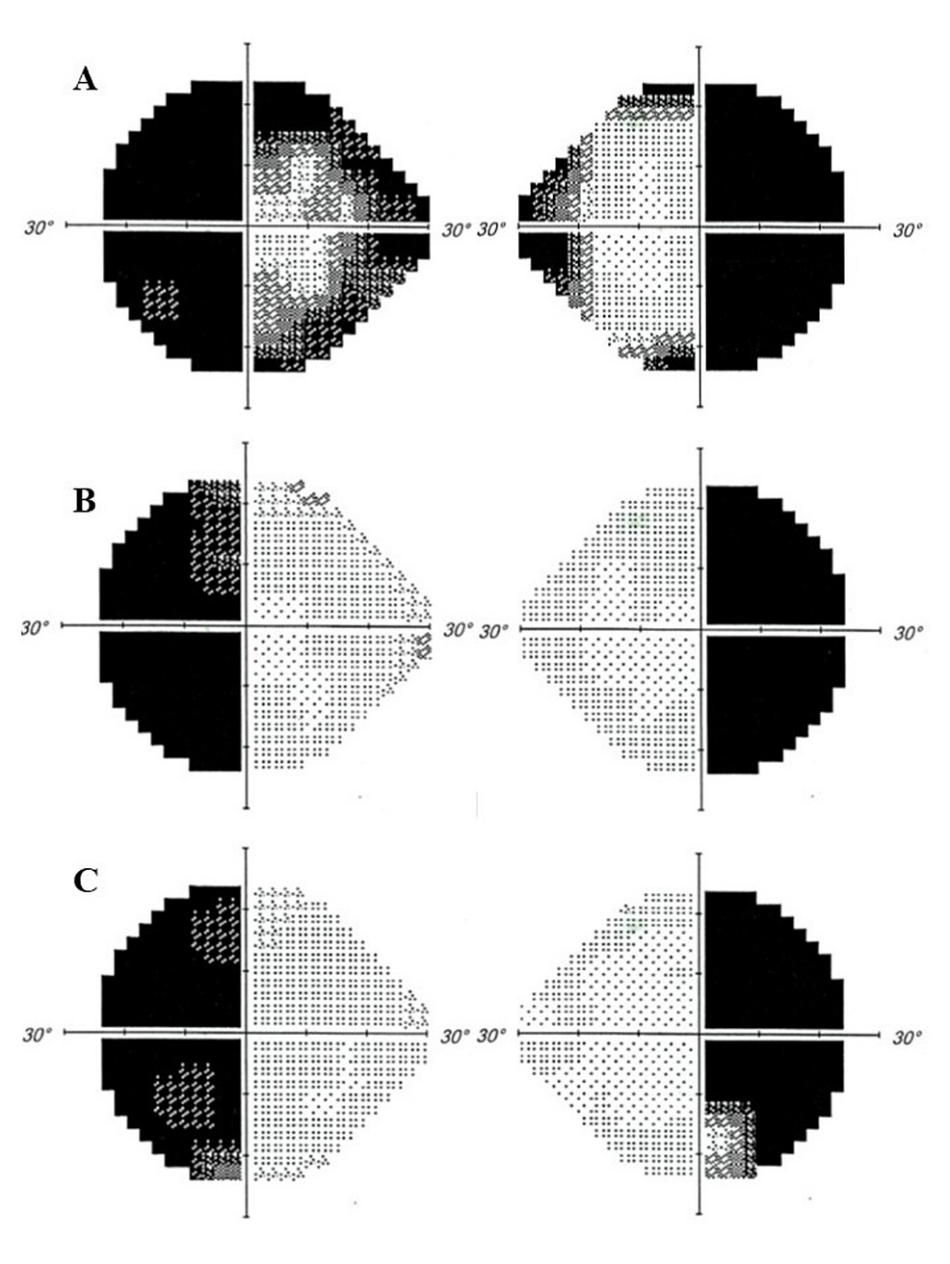
Serial Humphrey visual field perimetry showing bitemporal hemianopia. Serial Humphrey visual field perimetry confirming persistent visual field defect. The visual field was evaluated (A) one month post-trauma, (B) ten months later, and (C) 19 months later.

He was treated conservatively by ophthalmology and neurosurgical teams. Upon discharge, the right eye visual acuity was 6/60, the left eye was 6/6, and persistent bitemporal hemianopia. On subsequent follow-ups within a year, the visual acuity has improved to 6/6 on the right eye and 6/6 on the left eye. The ocular movement was normal in both eyes without diplopia. Bilateral optic discs showed mild pallor. Persistent bitemporal hemianopia was confirmed by serial Humphrey visual field perimetry (Figure 3).

There were no other neurological deficits detected.

## Discussion

Traumatic chiasmal injury is uncommon, and bitemporal hemianopia is the primary finding, similar to other chiasmal disorders [2,3,5]. It is concerning that more cases of chiasmal damage have not been recorded, given the rise in the number of head injuries involved in road traffic accidents. About 3.2% of head trauma patients with optic pathway involvement had chiasmal injury [6].

The mechanism of traumatic chiasmal injury varies from microtears, although rare, to complete transection of the chiasm following severe head injury [2,7]. The midline structures such as chiasmal fibers receive the maximum shearing forces from the frontal blow and cause sudden stretching of the fibers [8]. This also causes fine vessels to rupture and leads to chiasmal ischemia and later necrosis. Another postulation is due to contusion necrosis of the optic chiasm from contrecoup head injury [2]. These mechanisms can occur simultaneously.

The patients may present with a wide range of visual acuity from 6/6 to counting fingers irrespective of the severity of the head injury and visual field loss [3,5]. Asymmetric vision loss in both eyes is possible, and some cases of unilateral vision loss with temporal visual field deficits in the contralateral eye have been reported [2,3]. The onset of visual field loss is immediate but the detection is usually late as most patients require ventilatory support. Our patient showed improvement in visual acuity despite persistent bitemporal hemianopia similar to the previously reported case [9]. Previous studies found that patients with traumatic bitemporal hemianopia had frontal and anterior basal skull fractures associated with intracerebral and subarachnoid hemorrhage similar to our present case [2,5].

Bitemporal hemianopia is treated conservatively. Other neurological complications may arise, such as cranial nerves palsy, hypopituitarism, meningitis, carotid-cavernous fistula, and carotid aneurysm. Some authors reported the incidence of transient diabetes insipidus [3,4]. However, the current case demonstrates only isolated bitemporal hemianopia without endocrine problems or other neurological deficits.

## Conclusions

Traumatic bitemporal hemianopia is rarely encountered in ophthalmology practice. Late detection and diagnosis are possible as most patients sustained a severe head injury with varying duration of loss of consciousness and survival outcome. Therefore, the chiasmal injury should be considered as one of the associated injuries when dealing with frontal head trauma and midline facial fractures.
